# Tea and Recurrent* Clostridium difficile* Infection

**DOI:** 10.1155/2016/4514687

**Published:** 2016-08-29

**Authors:** Martin Oman Evans II, Brad Starley, Jack Carl Galagan, Joseph Michael Yabes, Sara Evans, Joseph John Salama

**Affiliations:** ^1^Department of Internal Medicine, Novosel Aviation Clinic, Fort Riley, KS, USA; ^2^Department of Gastroenterology, St. Luke's Clinic, Twin Falls, ID, USA; ^3^Department of Internal Medicine, San Antonio Military Medical Center, San Antonio, TX, USA; ^4^Physical Medicine and Rehabilitation, Larkin Community Hospital, South Miami, FL, USA; ^5^Novosel Aviation Clinic, Fort Riley, KS, USA

## Abstract

*Background and Aims*. Studies have shown effects of diet on gut microbiota. We aimed to identify foods associated with recurrent* Clostridium difficile* infection (CDI).* Methods*. In this cross-sectional survey, consecutive patients diagnosed with CDI were identified by electronic medical records. Colitis symptoms and positive* Clostridium difficile *assay were confirmed. Health-care onset-health-care facility associated CDI was excluded. Food surveys were mailed to 411 patients. Survey responses served as the primary outcome measure. Spearman's rank correlation identified risk factors for CDI recurrence.* Results*. Surveys were returned by 68 patients. Nineteen patients experienced CDI recurrence. Compared to patients without CDI recurrence, patients with CDI recurrence had more antibiotics prescribed preceding their infection (*p* = 0.003). Greater numbers of the latter also listed tea (*p* = 0.002), coffee (*p* = 0.013), and eggs (*p* = 0.013), on their 24-hour food recall. Logistic regression identified tea as the only food risk factor for CDI recurrence (adjusted OR: 5.71; 95% CI: 1.26–25.89).* Conclusion*. The present results indicate a possible association between tea and CDI recurrence. Additional studies are needed to characterize and confirm this association.

## 1. Introduction

The incidence of* Clostridium difficile *infection (CDI) is on the rise and has increased by a factor of three over the last decade [1]. In addition, the 30-day mortality rate for CDI is high [2] and the reported recurrence rate currently ranges from 15 to 20% [3]. However, in a recent study, the recurrence rate for CDI decreased after a duodenal infusion of feces was performed [4]. It is hypothesized that a fecal transplant reestablishes equilibrium in a microbiota by restoring specific bacterial species that are lacking. Recent nutrition studies have also demonstrated that diet reproducibly and predictably affects microbiota [5, 6]. Therefore, the aim of this study was to identify foods that may be associated with recurrent CDI in humans.

## 2. Methods

### 2.1. Study Design

Institutional review board approval (number 404471) was obtained to review medical records and mail questionnaires to appropriate patients according to a Health Insurance Portability and Accountability Act (HIPAA) waiver.

In this cross-sectional survey, electronic medical records from military clinics and corresponding regional hospitals were reviewed. Records that included an International Classification of Diseases (ICD) code-9 for CDI and a corresponding Current Procedural Terminology code for* C. difficile *assay were used to identify patients with CDI. The latter included an Xpert* C. difficile*/Epi Assay or an Xpert* C. difficile* Assay. Between October 2008 and December 2014, 986 consecutive patients were found to be treated for CDI. Each medical chart was reviewed individually to confirm colitis symptoms, positive assay, and baseline medical information. Patients with health-care facility-onset health-care facility associated (HO-HCFA) CDI, patients younger than 18 years of age, and patients who had died were subsequently excluded. Patients with HO-HCFA CDI were excluded because these patients were hospitalized for more than 72 h prior to CDI diagnosis and their in-hospital diet would not represent their at-home diet habits.

Therefore, 411 patients were mailed a food frequency and 24-hour food recall questionnaire. The food frequency questionnaire (FFQ) surveyed 19 food items patterned after the Diet History Questionnaire, a FFQ developed by the National Cancer Institute. This was the same format used by National Health and Nutrition Examination Survey III. The survey responses served as the primary outcome measure. The 24-hour food recall was performed only once. The FFQ asked participants to consider the past 12 months of food consumption. Subjects were asked to consider current diet habits. These surveys were mailed several months to years after the patients completed treatment or laboratory evaluation. The diet habits reflected would therefore be after any infection or recurrence of* C. difficile.*

The patients that returned the questionnaire were divided into two groups based on recurrent CDI. Patients with recurrent CDI were defined as those that had a resolution of their CDI symptoms prior to a recurrence of symptoms and also had a second positive* C. difficile *assay > 10 days and < 60 days after their initial positive assay.

## 3. Statistical Analysis

The Department of Clinical Investigation at San Antonio Military Medical Center analyzed the data. Baseline characteristics and questionnaire responses were analyzed. The method of Kraemer and Thiemann estimated that the sample size was adequate for a power of 80%. Spearman's rank correlation analysis was used to determine significant relationships. Baseline characteristics and food items with *p* values < 0.05 and correlation coefficient of 0.50 were then included in logistic regression performed with the Hosmer-Lemeshow test.

## 4. Results

Of the 411 patients contacted, 68 returned the survey provided and 19/68 (28%) had experienced CDI recurrence. Among the baseline characteristics included in our analysis (Table 1), prescription of non-*C. difficile* antibiotic 4 or 8 weeks prior to CDI diagnosis was found to be significantly associated with CDI recurrence. In particular, patients with CDI recurrence tended to have antibiotics prescribed within the 4 weeks preceding their infection compared with the patients without CDI recurrence (89% versus 51%, resp.; *p* = 0.003). Correspondingly, an analysis of the antibiotic prescription data by logistic regression indicated an odds ratio (OR) of 7.81 (95% confidence interval (CI), 1.30–46.8).

A comparison of the 24-hour food recall list for patients with and without CDI recurrence showed that consumption of tea (47% versus 14%, resp.; *p* = 0.002), coffee (53% versus 24%, resp.; *p* = 0.013), and eggs (47% versus 20%, resp.; *p* = 0.013) differed between the two groups (Table 2). However, the logistic regression performed identified mentioning tea as the only food risk factor for recurrent CDI (adjusted OR: 5.71; 95% CI, 1.26–25.89). In the logistic regression analysis performed mentioning coffee on 24-hour recall was not able to predict CDI recurrence (adjusted OR 4.154; 95% CI, 0.969–17.819). Additionally, mentioning egg was not able to predict recurrence (adjusted OR 4.380; 95% CI, 0.986–18.609). After two steps of backward elimination with the Hosmer-Lemeshow test, tea remained significant. This indicates that the observed distribution matched the expected distribution of our prediction model. Tea was the only food item able to predict CDI recurrence independent of antibiotic presence in our small study.

The FFQ responses also supported the results of the 24-hour recall responses. In the CDI recurrence group, 19 of 19 drank tea in the past year, compared with 41 of 48 in the group of patients without CDI recurrence (100% versus 85.4%, resp.; *p* = 0.044). The median frequency of tea consumption was also higher in the CDI with recurrence group (18 servings per month versus 6 servings per month; *p* = 0.020). The CDI with recurrence group showed a tendency to drink more coffee; 17 of 19 did so in the past year versus 38 of 49 in the group without recurrence (89.5% versus 77.6%, resp.; *p* = 0.106). The median consumption of coffee was higher in recurrence group (28 servings per month versus 22 servings per month; *p* = 0.015). Unfortunately egg consumption was not assessed on the FFQ.

## 5. Discussion

Of the 68 patients examined in the present study, those with CDI recurrence were more likely to list consumption of tea, coffee, and eggs in the 24-hour recall survey they returned. When a logistic regression model was applied, both antibiotic use four weeks prior to CDI development and consumption of tea (from 24-hour recall) were factors that predicted CDI recurrence. The results from the FFQ provided additional evidence of the association between tea consumption and CDI recurrence.

In the present study the power was limited to 80% due to sample size. This results in a large 20% risk of type two errors. It is certainly possible that other food items are identified in future studies.

FFQ and 24-hour surveys have been shown to correlate with serum markers of diet intake [7]. However, recall bias and misreporting in surveys are known phenomena [8]. The authors readily admit that the methodology in the present study is inferior to prospective studies in which food consumption is observed. Study design steps were taken to improve data quality. Firstly, the FFQ was patterned after a validated format developed by the National Cancer Institute [9]. Secondly, subjects were asked to recall current diet trends. Lastly, diet was assessed in two different manners, FFQ and 24-hour recall. These mitigations do not make this study preferable to a prospective trial with improved methodologies. The association between tea and CDI recurrence should be confirmed with studies of higher evidence. Additionally, future studies should be prospective, having more inclusive criteria and larger sample sizes.

In the present study both food measurement tools showed tea consumption differences between groups. Another recent study evaluated tea consumption. Their control group consisted of military veterans undergoing elective esophagogastroduodenoscopy or screening colonoscopy. They found 878/1728 (50.4%) were ever tea drinkers [10]. This is compared with our FFQ of the present study. Of those who had CDI without recurrence, 85.4% drank tea in the last year. Of those who had a CDI with recurrence, 100% drank tea in the last year.

It is hypothesized that the survey respondents consumed black tea more often than other types of tea since black tea is the type of tea most often consumed in the United States [11]. However, most respondents listed “tea” without specifying the type or temperature. While the production of black tea involves the partial oxidation of green tea, many components are similar in both tea varieties [11].

*C. difficile* is not considered a foodborne threat [12].* C. difficile* is considered ubiquitous; therefore exposure to this bacterium seems inevitable. Small studies have found that 2.3–7.5% of vegetable samples and 0–62.5% of meat samples contain* C. difficile*[12]. Additionally,* Clostridium *spp. have been found in unpasteurized tea [13]. However, the incidence of CDI is far lower, occurring at an average rate of 147 per 100,000 person-years [14]. Thus, very few of those exposed to* C. difficile* develop CDI.

The population that develops CDI after* C. difficile* exposure is likely unique. For example, they have been shown to have decreased immune response to* C. difficile* toxin among other things [15]. Those who develop CDI and CDI recurrence also may have higher intake of tea. This is suggested in the present study. Tea may be associated with CDI and CDI recurrence not by infecting patients but by promoting an environment where* C. difficile* may colonize and recur.

In a meta-analysis, continued antibiotic use that was not associated with* C. difficile *treatment was found to be the most consequential risk factor for CDI recurrence [3] (OR, 4.2; 95% CI, 2.10–8.55). Tea has also been shown to have antimicrobial effects, and this may explain the identification of tea consumption as a risk factor for CDI recurrence in the present study.

Tea has many individual components that have been shown to be active against human oral flora [16], pathogenic microbes* in vitro* [16], and commensal flora* in vitro* [17]. Most studies describing the antimicrobial activity of tea have been performed* in vitro* or in animal models. One* in vivo* study was performed in 13 healthy human volunteers and showed a decrease of 1.4 × 10^10^ bacterial cells per gram of wet feces when black tea was consumed [18]. It is likely that this tea's antimicrobial action occurs in the colon based on the results of a study performed with ileostomy patients where 70% of tea flavonols (both parent compounds and metabolites) were detected in the ileal fluid [19]. These results suggest that a large number of tea compounds reach the colon. However, given the paucity of human* in vivo *studies that have examined the effect of tea on microbes [20], many questions remain unanswered.

There are various possible explanations of the current study. It is certainly possible that tea has a causal relationship with CDI recurrence. However that conclusion is beyond the scope of the present study. It is important to note that the food surveys were filled out months to years after patients recovered and were no longer under treatment or laboratory evaluation. As a result, it is possible that diet changes were made after the CDI or CDI recurrence. While a healthy diet has been encouraged in patients with CDI, there is no consensus recommendation regarding what foods to ingest or avoid [21, 22]. The Infectious Disease Society of America identified nutrition as a “research gap” in the colonization of* C. difficile* [23]. It is therefore unlikely that our participants were given consistent or specific advice. They were likely told to eat healthier and interpreted this in their own manner. Those with CDI recurrence may be more likely to begin consuming tea. That is a possible explanation of the present study. Additionally epigallocatechin gallate, an isolate of green tea, was recently shown to suppress virulence and was bactericidal in mice with CDI [24]. The association of tea and CDI recurrence could be causative or curative. Lastly, it should be noted that there was no asymptomatic control group in the present study. Certainly a great majority of persons consume tea without ever developing CDI because the absolute risk of CDI is so low. The present study cannot determine if tea consumption increases one's relative risk for CDI.

In the present study, consumption of tea was identified as a risk factor for predicting CDI recurrence in our logistic regression model. Previously identified risk factors for CDI recurrence have included continued use of non-*C. difficile* antibiotics, age > 75 years, renal failure, and antiulcer medications [3]. In the present study, 15 patients were older than 75 years, 8 patients had a creatinine level greater than 1.5, and 16 patients were on antiulcer medication. It is not clear why age, renal failure, and antiulcer medication were not identified as significant risk factors in the present study, although the exclusion of HO-HCFA CDI patients may have played a role. In addition, because the chart reviews were performed retrospectively, a high quality metric to assess whether the participants continued non-*C. difficile* antibiotics was not able to be established. Rather pharmacy records were reviewed to determine which patients had access to non-*C. difficile* antibiotics.

## 6. Conclusion

The results of the present study indicate a possible association between CDI recurrence and diet, particularly in regard to the consumption of tea, coffee, and eggs. These data are consistent with the growing body of evidence suggesting that diet affects the microbiota. However, further studies are needed to characterize and confirm the association of tea with CDI recurrence.

## Figures and Tables

**Table 1:** Characteristics of the study population.

Characteristics of the study population^a^	Recurrence (*N* = 19)	No recurrence (*N* = 49)	*p* value
Male, *N* (%)	9 (47)	21 (43)	0.61
Age (years), median (IQR)	62 (55, 72)	63 (51, 74)	0.73
BMI (kg/m^2^), median (IQR)	27.8 (25, 30.8)	26.8 (23.6, 30.9)	0.32
IFG or diabetes, *N* (%)	2 (11)	9 (18)	0.44
WBC^b^(×10^3^/mm^3^), median (IQR)	10.3 (7.1, 12.5)	11.30 (6.6, 12.8)	0.95
Creatinine^b^ (mg/dL), median (IQR)	0.90 (0.80, 1.01)	0.97 (0.78, 1.38)	0.43
Albumin (g/dL), median (IQR)	3.45 (2.95, 4.40)	3.50 (3.10, 4.10)	0.92
Antibiotic prescribed within 8 weeks prior to CDI, *N* (%)	18 (95)	35 (71)	**0.038**
Antibiotic prescribed within 4 weeks prior to CDI, *N* (%)	17 (89)	25 (51)	**0.003**
Prescribed proton pump inhibitor, *N* (%)	2 (11)	10 (20)	0.35
Prescribed H2 blocker, *N* (%)	1 (5.3)	4 (8.2)	0.69
Admitted to hospital, *N* (%)	12 (63)	25 (51)	0.38
Days of hospitalization, median (IQR)	3.5 (3.50, 5.25)	4.0 (3.0, 4.0)	0.59
Initial treatment with metronidazole, *N* (%)	16 (84)	41 (84)	0.50
Initial treatment with vancomycin, *N* (%)	2 (11)	8 (16)	0.33
Initial treatment with metronidazole and vancomycin, *N* (%)	1 (5.3)	0 (0)	0.054

^a^Missing values: WBC = 14, creatinine = 12, and albumin = 15.

^b^The highest value if more than one value present.

**Table 2:** Foods mentioned in 24-hour recall listed by prevalence.

Food mentioned	Number of participants mentioning food		*p* value
	Recurrence group (%)	No recurrence group (%)	
Fried items	7 (37)	24 (49)	0.816
Chicken	8 (42)	17 (35)	0.285
Cheese	7 (37)	18 (37)	0.497
Breads	9 (47)	16 (33)	0.129
Coffee	10 (53)	12 (24)	**0.013**
Eggs	9 (47)	10 (20)	**0.013**
Yogurt	6 (32)	12 (24)	0.276
Tea	9 (47)	7 (14)	**0.002**
Tomatoes	6 (32)	9 (18)	0.119
Water	4 (21)	11 (22)	0.550
Fish	3 (16)	8 (16)	0.522
Salad	7 (37)	9 (18)	0.054
Potatoes	4 (21)	12 (24)	0.618
Broccoli	6 (32)	7 (14)	0.052
Milk	2 (11)	11 (22)	0.869
Sandwich	4 (21)	11 (22)	0.550
Banana	5 (26)	7 (14)	0.121

## Abbreviations

CDI: *Clostridium difficile* infection
HO-HCFA: Health-care facility-onset health-care facility associated
FFQ: Food frequency questionnaire
CI: Confidence Interval.
